# Online Game Speculative Experiences and Problem Gambling among Adolescents in South Korea: Dual Mediation Effects of Irrational Beliefs and Gambling Attitude

**DOI:** 10.3390/healthcare11091226

**Published:** 2023-04-25

**Authors:** Jae-Kyoung Lee, Soo-Bi Lee

**Affiliations:** 1 Department of Child Studies & Social Welfare, Andong National University, Andong-si 760-749, Republic of Korea; 2 Division of Social Welfare and Child Studies, Daejin University, Pocheon-si 487-711, Republic of Korea

**Keywords:** adolescents, betting game, online game speculative experience, gambling attitude, irrational belief, problem gambling

## Abstract

This study aimed to test the effect of online gaming speculative experiences on problem gambling via irrational beliefs in and attitudes toward gambling. Data were obtained from the Korea Center on Gambling Problems, and participants comprised 386 adolescents (female 168, male 218) who currently play online games and have experience with betting games or gambling. The main findings are that (i) online game speculative experience positively influenced gambling attitude (*B* = 0.172, *p* < 0.001); (ii) online game speculative experience positively influenced irrational beliefs (*B* = 0.194, *p* < 0.001); (iii) online game speculative experience (*B* = 0.140, *p* < 0.001), gambling attitude (*B* = 0.294, *p* < 0.01), and irrational beliefs (*B* = 0.689, *p* < 0.001) was positively correlated with problem gambling. Also, the mediation effect was statistically significant. Policy and practical measures to assess the impact of gaming facilitating speculative experience and for intervening in gambling problems in adolescents are discussed. The results suggest the need to screen, educate, and provide short-term interventions to adolescents with online game speculative experience. Strict assessments, regulation, and surveillance of speculative elements can preserve online gaming as a healthy play culture for the adolescents.

## 1. Introduction

Approximately 90% of the population of adolescents in South Korea report playing online games. They engage in online gaming at least 4–5 times per week using a mobile phone, and at least 2–3 times per week using a PC. This suggests that playing online games is a common leisure activity among South Korean adolescents. The number of adolescents engaging in online gaming in South Korea has further increased particularly since the COVID-19 pandemic [1]. Therefore, potential harms likely to arise in adolescents due to online gaming have become a social issue in South Korea. Recently, there have been media reports not only of gaming addiction, but also problems such as adolescents using their parents’ credit cards to secretly buy in-game items, or gambling to get money to play games (https://www.yna.co.kr/view/AKR20220601042800017, accessed on 23 April 2023).

Adding speculative elements in online gaming, such as “gaming loot boxes” (in which the effects and functions of purchasable items for in-game convenience are redeemed based on chance), is a business model used by many online gaming companies to generate profits [2]. Although online gaming speculative elements look like “just a game” on the outside, many researchers in essence equate it to gambling because it generates chance-based outcomes of extrinsic rewards and expenditures [3,4,5,6,7,8,9].

Based on Blaszczynski and Nower’s [10] pathways model of problem and pathological gambling, the online game speculative experience is assumed to be an important variable for access to gambling. Gambling behavior and problems related to gambling are developed through access and exposure to gambling [11], either directly [12] or indirectly, via irrational beliefs or attitudes toward gambling [10,13,14,15,16,17]. In this context, online game speculative experience indirectly provides game users with an experience similar to gambling, promoting gambling behavior or causing problem gambling in the future by playing the role of a gateway to gambling problems or increasing irrational beliefs, curiosity, and receptive attitudes toward gambling [18,19,20]. Based on previous study findings, online game speculative experience may therefore be considered a starting point and a strong predictor of problem gambling.

Studies on the online game speculative elements and gambling problems were conducted with a focus on “gaming loot boxes” purchase experience, purchase frequency, and risky use [9,20,21,22,23,24,25]. As the frequency and duration of using gaming loot boxes and the expenditure increased, gambling problems became more serious [9,25].

Recently, however, game users have been exposed to online game speculative elements in a variety of forms and manners, including not only the experience of purchasing loot boxes in online games but also the exposure to information regarding redeemable gaming loot boxes (which resembles “the exposure to a jackpot won in gambling”), and direct and indirect exposures to “big-win” and “near-miss” experiences pertaining to the items. Additionally, “minigames” were added, i.e., short games such as lottery, card games, and races provided separately within an online game and played by betting in-game items or currency [26]. Furthermore, “the opening of a temporary gambling board like the odds and evens game played with in-game currency or items among game users” and “advertisements for illegal online gambling sites in in-game chats or utilizing game characters” are found in online games [4]. However, many previous studies are limited in that they investigated online game speculative elements by focusing only on the “gaming loot box” purchase experience and respective frequency.

Studies conducted in South Korea reported that direct and indirect online game speculative experiences, such as big-win and big-loss experiences in online gaming [27], exposure to information on the winning of others [28], and gaming loot box purchase and relevant content viewing [5], negatively affect gambling attitude and problems. These findings highlight the need to investigate online game speculative experiences more specifically and in a multifaceted manner, rather than by examining simple use, frequency, and duration of online games.

In South Korea, the estimated rate of gambling among risk groups, i.e., 6.4% [29], is higher than the prevalence of gambling addiction in adults, i.e., 5.3%. Adolescence is a period in which identity is formed; it is a critical developmental stage both physically and mentally because the value system and habits developed during this period exert significant influences in adulthood [30]. Many adolescents often encounter online games due to peer relationships or as a means of coping with stress, and often encounter speculative experiences in games. Also, the adolescence is highly vulnerable to addictive stimuli because in adolescence, the prefrontal function of controlling the reward circuit is not fully developed [31,32]. Therefore, it is necessary to pay attention to the experience of gambling in online games among adolescents.

Accordingly, this study aimed to assess the online game speculative experiences of adolescents (i.e., primary online game users) in a multifaceted manner and more specifically than simply examining the pattern of use and to explore the pathway to irrational beliefs and gambling attitude in order to understand the impact of online game speculative experience on problem gambling. An additional purpose of the study was to explore, based on study findings, clinical implications and interventions to prevent problem gambling.

## 2. Materials and Methods

### 2.1. Participants

This study utilized the raw data from the “study on online gambling, internet gaming, and facilitators of gambling” conducted by the Korea Center on Gambling Problems [4]. According to the research report, the survey was conducted for 12 days (14–26 October 2019) with people aged between 14 and 69 years. The sample was obtained using a quota sampling technique, controlling for age and sex, based on the 2019 statistics on the resident registration population available at the Korea Ministry of the Interior and Safety [4]. In this study, however, the data of 386 online game users aged between 14 and 19 years were extracted and analyzed since the analysis targeted adolescents.

However, since the subject of analysis in this study is only adolescents, the age was limited to 14 to 19 years old by applying the legal adolescent age standard of Korea, and among them, adolescents using online games were extracted and a total of 386 people were used for analysis.

### 2.2. Measures and Procedure

#### 2.2.1. Dependent Variable: Problem Gambling

To measure problem gambling, the Gambling Problems Severity Scale (GPSS) from the Canadian Adolescent Gambling Inventory (CAGI) was used [33]. This scale has been adapted by the Korea Gambling Problem Management Center and is being used in the Korean language [34]. The GPSS consists of 9 items that are rated on a 4-point Likert scale (0–3 points), assessing experiences in the last 3 months. Based on cut-offs, the scores are interpreted as no problem gambling (0–1 point), low-to-moderate severity (2–5 points), or high severity of problem gambling (6 points or higher). In this study, however, the GPSS score was treated as a continuous variable. The higher the total score (range: 0–12 points), the more severe the problem gambling. Cronbach’s α was 0.95 in the present sample.

#### 2.2.2. Independent Variable: Online Game Speculative Experience

The online game speculative experience was assessed using the “Internet Game Speculative Experience”, a self-report scale developed by the Korea Center on Gambling Problems (a public institution providing services regarding gambling problems) and Kwon et al. [13] to measure the level of online game speculative experience in game users. It consists of 10 items focusing on online game speculative experience, including the following: “I have purchased gaming loot boxes or collected characters that I can use at any time”, “I have purchased gaming loot boxes or collected characters during a paid event (a limited time)”, “I have played minigames within a game which was like gambling”, “when collecting items, I have the experience of unfortunately missing an opportunity to get high-priced (rare) items or characters”, “I have participated in a gambling board in which users play by betting in-game currency or items”, “I have lost items or characters while trying enhancement or synthesis”, and “I have seen characters promoting a betting game on the toto site”. The items are rated on a 4-point Likert scale with the anchors “never (0) –every time (3)”. In this study, a total score was obtained by summing the 10 items (range: 0–30 points), which was treated as a continuous variable. The higher the total score, the higher the level of online game speculative experience. Cronbach’s α was 0.87 in the present sample.

#### 2.2.3. Mediating Variables: Gambling Attitude, Irrational Belief

Attitude toward gambling was assessed using 5 items regarding “positive attitude toward gambling” selected by the Korea Center on Gambling Problems [4] and Kwon et al. [13] from 35 items in the Korean version of the Gambling Attitude and Beliefs Scale (K-GABS). The instrument was originally developed by Been and Zuckerman (1994) to measure vulnerability to gambling problems. The 5 items are “Gambling makes me feel really alive”, “I respect a person who makes very large bets and remains calm and cool)”, “People who gamble are more daring and adventurous than those who never gamble”, “If I were feeling down, gambling would probably pick me up”, and “I like gambling because it helps me to forget my everyday problems”. The items were rated on a 4-point Likert scale with anchors ranging from “never (0 point)” to “almost always (3 points)”. The higher the total score obtained by summing all items, the more positive the attitude toward gambling. Cronbach’s α was 0.74 in the present sample.

Irrational beliefs were assessed with the irrational gambling belief scale developed by Kim and Kwon [35]. The scale consists of the following 4 items: “good strategies should be developed to win in betting games and gambling”, “In betting games and gambling, it is better to have a few big wins than have several small wins”, “In betting games and gambling, the probability of winning increases if previous outcomes are referred to”, and “if there is a debt while playing a betting game or gambling, it is the best to repay by winning it”. The items were rated on a 4-point Likert scale (0 point for “never” to 3 points for “almost always”). The higher the total score, the stronger the irrational belief in gambling. Cronbach’s α was 0.72 in the present sample.

#### 2.2.4. Control Variables

Sex (male = 1, female = 2), age (continuous variable), and gaming frequency (measured on a 4-point Likert scale with a value of 1 for “currently not”, 2 for “sometimes”, 3 for “do it often” and 4 for “daily”) were treated as control variables.

### 2.3. Statistical Analysis

Data were analyzed using SPSS 27 and PROCESS macro 3.5. First, participants’ general characteristics and correlations between variables were examined by performing frequency analysis, descriptive statistics, and correlational analysis. Second, to test the mediation effects of irrational beliefs and gambling attitudes on the effect of online game speculative experience on problem gambling, multiple regression analysis was performed using PROCESS macro Model 4. Lastly, the statistical significance of the mediation effects was tested by generating 5000 bootstrap samples and computing 95% confidence intervals [36].

### 2.4. Ethics

All procedures performed in this study were in accordance with the ethical standards of the institutional research committee and with the 1964 Helsinki Declaration and its later amendments. Informed consent was obtained from all individual participants involved in the study [IRB: KYU-2019-284-02].

## 3. Results

### 3.1. General Characteristics of Participants and Descriptive Statistics of Primary Variables

Participants’ general characteristics are shown in Table 1. A total of 386 participants were included, of whom 56.5% were male and 43.5% were female. Regarding gaming frequency, 33.2% responded with “often”, 31.6% with “daily”, and 30.3% with “sometimes”. Mean age was 18 years (SD ± 1.4). The score for online game speculative experience (independent variable) ranged 0–30 points, with a mean of 10.3 (SD ± 6.3). Scores for gambling attitude (mediating variable) ranged from 0 to 15, with a mean of 4.5 (SD ± 2.8); scores for irrational belief ranged from 0 to 12, with a mean of 3.4 (SD ± 2.8). Scores for problem gambling (dependent variable) ranged from 0 to 12, with a mean of 3.0 (SD ± 5.1). The absolute values of skewness and kurtosis exceeded 3 and 8, respectively, or 2.1 and 7.1, respectively, for any of the primary variables [37,38]. In this study, the skewness and kurtosis of speculative experience were 0.46, −0.24, the skewness and kurtosis of gambling attitude were 0.68, 0.31, the skewness and kurtosis of irrational belief were 0.66, −0.02, and the skewness and kurtosis of problem gambling was 1.15, 0.94. Thus, the normality assumption was met.

### 3.2. Testing the Research Model

To examine multicollinearity between the primary variables, correlational coefficients were computed. Absolute values of the coefficients were 0.329–0.469 and did not exceed 0.8; accordingly, we determined that there was likely no issue of multilinearity. In the analysis conducted using PROCESS macro Model 4 to test the mediation effects of irrational beliefs and gambling attitudes on the online game speculative experience on problem gambling, the following results were obtained (Table 2). First, in step 1, regression analysis was performed including online game speculative experience (independent variable) and gambling attitude (mediating variable 1) as the dependent variable, adjusted with control variables. The resulting model fit was statistically significant (*F* = 20.887, *p* < 0.001), and online game speculative experience positively (+) influenced gambling attitude (*B* = 0.172, *p* < 0.001). In contrast, sex was negatively associated with problem gambling (*B* = −0.727, *p* < 0.010).

In step 2, the effect of online game speculative experience (independent variable) on irrational beliefs in gambling (mediating variable 2) was tested. The resulting regression model was statistically significant (*F* = 25.681, *p* < 0.001), and online game speculative experience positively influenced irrational beliefs (*B* = 0.194, *p* < 0.001).

In the last step (step 3), regression analysis was performed to test the effects of online game speculative experience, gambling attitude, and irrational beliefs on problem gambling. Again, model fit was statistically significant (*F* = 37.172, *p* < 0.001), and the effects of independent variables (i.e., online game speculative experience (*B* = 0.140, *p* < 0.001), gambling attitude (*B* = 0.294, *p* < 0.01), and irrational beliefs (*B* = 0.689, *p* < 0.001)) were positively statistically significant. In addition, sex (*B* = −0.939, *p* < 0.05) and gaming frequency (*B* = −0.822, *p* < 0.001) were negatively associated with problem gambling.

**Figure 1:**
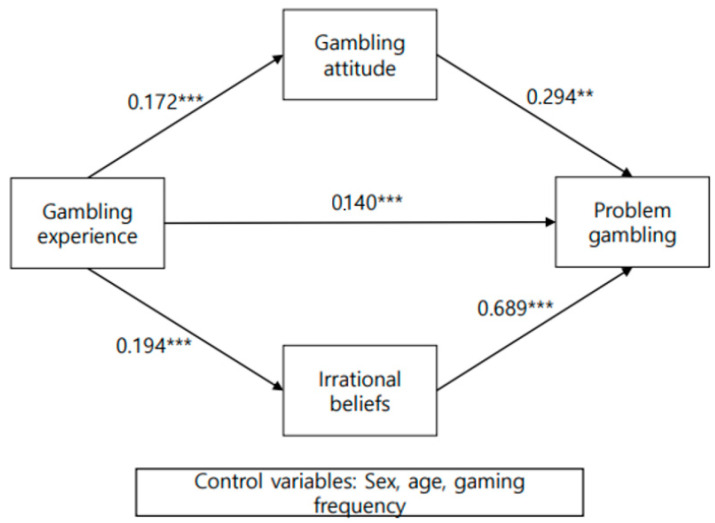
Research model result. Notes. ** *p* < 0.01. *** *p* < 0.001.

The outcomes of testing the aforementioned research hypotheses are visualized below (Figure 1). Online game speculative experience was demonstrated in the study to foster positive attitudes toward gambling, increase irrational beliefs in gambling, and ultimately, influence problem gambling in adolescents.

Next, mediation effects of gambling attitudes and irrational beliefs on the relationship between online game speculative experience and problem gambling were tested (Table 3). First, the direct effect of online game speculative experience on problem gambling (*B* = 0.140) was found to be statistically significant (t = 3.524, *p* < 0.001). Second, the mediation effect of online game speculative experience on problem gambling via gambling attitude was also statistically significant. The size of the mediation effect was 0.051 and the 95% confidence interval did not contain 0, thus confirming statistical significance of the mediation effect (95% CI: 0.013~0.090). Third, the mediation effect of online game speculative experience on problem gambling via irrational belief was 0.134 and statistically significant (95% CI: 0.081~0.192).

## 4. Discussion

The study aimed to test the effect of online gaming speculative experiences on problem gambling via irrational beliefs in and attitudes toward gambling. The main findings were as follows. First, the online game speculative experience had a direct effect on problem gambling in adolescents. This finding supports the findings of several previous studies, which reported, from the viewpoint of access to gambling, that the experience of purchasing gaming loot boxes influenced gambling behavior [5,9,25,27,28]. The findings also seem to be in line with another study, where online game speculative behavior was shown to be harmful because it resembles gambling [39,40]. Second, we found that as online game speculative experience increased, attitude toward gambling became more positive, and irrational beliefs in gambling became stronger, ultimately influencing problem gambling. Additionally, direct and partial mediation effects of gambling attitude and irrational beliefs in the relationship between online game speculative experience and problem gambling were discovered. This finding supports a previous study where online game speculative experience created cognitive errors regarding gambling and influenced the formation of attitudes toward gambling [41]. From the viewpoint of access to gambling, this finding also supports the study finding that direct and indirect exposures to gambling and the environment mediated irrational beliefs in gambling, and influenced gambling behavior and gambling addiction in adolescents [42,43]. This study finding can be seen as consistent with previous findings that a group of adolescents who played online games showed a stronger intention to engage in gambling in the future compared to a group who did not [27] and that in adolescents with no gambling experience, the experience of purchasing gaming loot boxes positively affected the intention to participate in speculative activities in adulthood [16]. The previous and current study findings can be regarded as demonstrating that online game speculative experience, similar to gambling, helps form positive attitudes toward gambling as well as irrational beliefs, ultimately influencing problem gambling. According to social learning theory [44], which is widely known in the field of behavioral development, observation and vicarious experiences are influential. In this regard, the likelihood for adolescents to develop a positive perception of, and permissive attitude toward, speculative activity as they encounter the contents of speculative nature in online gaming frequently and in the long term may be considered as well.

The present findings have the following clinical and policy implications. First, the scope of prevention of gambling problems in adolescents and the offering of short-term interventions should be expanded. In particular, adolescents are highly likely to underreport gambling problem and behavior since they know that gambling is illegal and not acceptable by social norms. As a way to indirectly gauge the likelihood and also to pre-emptively evaluate the level of risk for gambling, assess thoroughly whether individual game players without the experience of gambling have online game speculative experience would be necessary. Adolescents with a high level of online game speculative experience should be considered as a potential gambling risk group and attentively monitored; additionally, they should be provided with preventive education consisting of diverse contents pertaining to speculative activities and gambling problems. Moreover, providing short-term intervention should be considered for those with a serious level of problem gambling.

Second, with respect to online games used by adolescents, in-game speculative elements and content should be strictly evaluated and regulated through consensus among stakeholders such as the gaming industry and the Korea Game Rating and Administration Committee. Currently, online games contain not only gaming loot boxes but also a wide variety of contents of speculative nature. In this respect, to prevent the adolescents from being exposed to speculative elements, in-game speculative features and contents should be evaluated thoroughly, strictly, and multidimensionally and real-time surveillance and management systems should be implemented in online games. From a public health perspective, in this effort, the duty to protect the health and daily lives of adolescents should precede industry logic.

This study focused on online game speculative elements, unlike previous studies, which typically focused on gaming use disorder, and investigated the path relationship with gambling attitude, irrational belief, and problem gambling. The study is of significance in that it demonstrated that as online game speculative experience increases, positive attitudes toward gambling and irrational beliefs also increase, ultimately influencing problem gambling, i.e., a behavioral addiction. Nonetheless, this study has limitations as it was a cross-sectional study conducted with adolescents; thus, investigating a change in problem gambling across time was not possible. In the future, longitudinal or follow-up studies should be conducted to examine the change in the severity of problem gambling in adulthood. The current discussion regarding how online game speculative experience influences gambling addition (a behavioral addiction) would thereby be further enriched.

In addition, this study is a study to confirm the effect of gambling experience through online games on adolescents, and there are limitations in measuring financial motivation, which can be understood as a major mechanism of problem gambling, such as winning money with online games. there is Therefore, in order to supplement the limitations of limiting gambling behavior to only the experience of gambling, an integrated study that considers other factors that are pointed out as causes of gambling behavior is proposed as a follow-up study.

## 5. Conclusions

Online gaming is a leisure activity and play culture enjoyed by adolescents worldwide. Particularly, in South Korea, more than 90% of the adolescents enjoy this leisure activity. Recently, however, its speculative nature, such as gaming loot boxes, became controversial. This study investigated the impact of online game speculative experience in diverse forms and contents on problem gambling and the mediation effects of primary predictors of gambling behavior, i.e., gambling attitude and irrational beliefs. It was found that as the level of online game speculative experience increased, positive attitudes toward gambling and irrational beliefs increased as well, and ultimately, the level of problem gambling increased. The present finding shows that online game speculative experience may function as a mechanism for reinforcing gambling behavior in adolescents. Based on the present finding, screening, educating, and providing short-term interventions to adolescents with online game speculative experiences are necessary. Additionally, strict assessments, regulations, and surveillance of speculative elements are necessary to preserve online gaming as a healthy play culture for the adolescents.

## Figures and Tables

**Table 1:** Descriptive statistics of the present sample.

Categorical Variables	Categories	Counts (n)	%
Sex	Female	168	43.5
	Male	218	56.5
Continuous variables	M	SD	Min~Max
Age	18	1.4	14–19
Gaming frequency	2.9	0.9	1–4
Speculative experience	10.3	6.3	0~30
Gambling attitude	4.5	2.8	0~15
Irrational belief	3.4	2.8	0~12
Problem gambling	3.0	5.1	0~12

Notes. N = 386. M = mean, SD = standard deviation, Min = minimum, Max = maximum.

**Table 2:** Mediation effects of gambling attitude and irrational beliefs on the relationship between online game speculative experience and problem gambling.

Categories	Step 1. Online Game Speculative Experience (x) → Gambling Attitude (M1)			Step 2. Online Game Speculative Experience (x) → Irrational Belief (M2)			Step 3. Online Game Speculative Experience (x) Gambling Attitude (M1), Irrational Belief (M2) → Problem Gambling (Y)		
	B	SE	95% CI	B	SE	95% CI	B	SE	95%CI
Online game speculative experience (x)	0.172 ***	0.022	0.128~0.216	0.194 ***	0.021	0.152~0.236	0.140 ***	0.039	0.062~0.218
Gambling attitude (M1)							0.294 **	0.089	0.118~0.470
Irrational belief (M2)							0.689 ***	0.093	0.506~0.873
Sex	−0.727 **	0.280	−10.278~−0.176	−0.405	0.269	−0.935~0.123	−0.939 *	0.448	−1.820~−0.059
Age	−0.038	0.096	−0.227~0.150	−0.024	0.092	−0.206~0.156	−0.041	0.152	−0.341~0.257
Gaming frequency	−0.224	0.153	−0.526~0.077	−0.103	0.147	−0.393~0.185	−0.822 ***	0.244	−10.301~−0.343
Constant	5.13 **	1.84	1.502~8.764	2.741	1.772	−0.744~6.227	2.410	0.370	−3.400~8.221
Model fit	R^2^ = 0.179, F = 20.887 ***			R^2^ = 0.212, F = 25.681 ***			R^2^ = 0.370, F = 37.172 ***		

Notes. N = 386. B = coefficient, SE = standard error of the coefficient, CI = confidence interval.* *p* < 0.05 ** *p* < 0.01. *** *p* < 0.001.

**Table 3:** Significance tests of total, direct, and indirect effects.

Categories		Effect	SE	t	P
Total effect	Direct + indirect effects	0.324	0.039	8.149	0.000
Direct effect	Online game speculative experience → problem gambling	0.140	0.039	3.524	0.000
		**Effect**	**BootSE**	**Boot LLCI—ULCI**	
Indirect effects	Online game speculative experience → Gambling attitude → Problem gambling	0.051	0.019	0.013–0.090	
	Online game speculative experience → irrational belief → Problem gambling	0.134	0.028	0.082–0.192	
	Total mediation effect	0.184	0.028	0.130–0.243	

Notes. Number of bootstrap samples = 5000. SE = standard error, BootSE = bootstrap standard error, BootLLCI = bootstrap lower limit confidence interval, BootULCI = bootstrap upper limit confidence interval.

## Data Availability

Not applicable.

## References

[1] Korea Creative Content Agency (2021). White Paper on Korean Games.

[2] Lee J.H., Yoo B. (2016). Impact of the introduction of gaming loot boxes on gamers’ loot box purchase and gaming imbalance. KMIS Int. Conf..

[3] Jung H.S. (2014). A critical review of inclination to gambling on game-items obtained by random logic. Chonnam Law Rev..

[4] Korea Center on Gambling Problems (2019). Study on Online Gambling and Facilitators of Internet Game Speculative Experience.

[5] Kwon S.J., Kim Y. (2018). Relationships among gaming loot box purchase, loot box combination, relevant content viewing, and gambling problem in the youth. Korean Psychol Assoc Annu. Conf. Program Abstr..

[6] Lee J.H. (2006). The criminal responsibility of gaming machine (AWP). Chung_Ang Law Rev..

[7] Lee R.H., Chang H.L., Lee J.K. (2018). The influences of winning money and gambling environments on problem gambling among adolescents in South Korea: Focusing on gender differences. J. Korea Contents Assoc..

[8] Ryu H. (2017). A study on the regulation on the game’s speculativeness in criminal cases. Inst. Leg. Stud. Chonnam Natl. Univ..

[9] Zendle D., Cairns P. (2018). Video game loot boxes are linked to problem gambling: Results of a large-scale survey. PLoS ONE.

[10] Blaszczynski A., Nower L. (2002). A pathways model of problem and pathological gambling. Addiction.

[11] Laplante D.A., Shaffer H.J. (2007). Understanding the influence of gambling opportunities: Expanding exposure models to include adaptation. Am. J. Orthopsychiatry.

[12] Adams G.R., Sullivan A.M., Horton K.D., Menna R., Guilmette A.M. (2007). A study of differences in Canadian university students’ gambling and proximity to a casino. J. Gambl. Issues.

[13] Kwon S.J., Kim Y., Kim E. (2020). Development of internet game speculative experience scale. Korean J. Health Psychol..

[14] Ladouceur R., Walker R., Salkovskis P.M. (1996). A cognitive perspective on gambling. Trends in Cognitive and Behavioral Therapies.

[15] Lee H.P. (2002). The Relationship of Irrational Gambling Belief, Gambling Motive, and Risk Taking with Pathological Gambling.

[16] Lee J.K., Lee R.H., Chang H.L. (2020). The association between modes of access to betting games and problem gambling among adolescents: Focusing on comparisons between on-line and land-based modes. J. Korea Contents Assoc..

[17] Yang J.N., Choi E.J., Lee M.H., So Y. (2011). Depression, impulsive behaviour and family health factors affects irrational belief of gambling and gambling behaviour of young people. Korean J. Youth Stud..

[18] Kwon S.J., Kim K.H., Choi J.O. (2006). Awareness of adult gambling and predictors of gambling behavior in children. Korean J. Health Psychol. Health.

[19] Kwon S.J., Kim K.H., Seong H.G., Rhee M.K., Kang S.G. (2007). Illegal internet gambling: Problems, risk factors, and prevention strategies. Korean J. Health Psychol. Health.

[20] Li W., Mills D., Nower L. (2019). The relationship of loot box purchases to problem video gaming and problem gambling. Addict. Behav..

[21] Brooks G.A., Clark L. (2019). Associations between loot box use, problematic gaming and gambling, and gambling-related cognitions. Addict. Behav..

[22] Hing N., Rockloff M., Russell A.M.T., Browne M., Newall P., Greer N., King D.L., Thorne H. (2022). Loot box purchasing is linked to problem gambling in adolescents when controlling for monetary gambling participation. J. Behav. Addict..

[23] Macey J., Hamari J. (2019). eSports, skins and loot boxes: Participants, practices and problematic behaviour associated with emergent forms of gambling. New Media Soc..

[24] Montiel I., Basterra-González A., Machimbarrena J.M., Ortega-Barón J., González-Cabrera J. (2022). Loot box engagement: A scoping review of primary studies on prevalence and association with problematic gaming and gambling. PLoS ONE.

[25] Zendle D., Cairns P. (2019). Loot boxes are again linked to problem gambling: Results of a replication study. PLoS ONE.

[26] Hwang J. [Special Topic Series ‘Popular’ Minigames in Online Game]. Too Dazzling ‘Minigames’,” Kyunghyang Games. https://www.khgames.co.kr/news/articleView.html?idxno=3125.

[27] Choi Y., Yoon H.Y. Influence of big-win/big-loss experiences in gambling on gambling attitude and behavior. Proceedings of the Korean Psychol Assoc Annual Conference Program & Abstract.

[28] Yang K.H., Kwak H.W., Chang M.S., Koo B.H. (2012). The effects of irrational gambling belief and reports of wins on gambling behavior. Korean J. Health Psychol. Health.

[29] Korea Center on Gambling Problems (2018). Survey on Youth Gambling Problems.

[30] Erikson E.H. (1998). The Life Cycle Completed (Extended Version).

[31] Derevensky J.L., Gupta R. (2000). Prevalence estimates of adolescent gambling: A comparison of the SOGS-RA, DSM-Ⅳ-J, and the GA 20 questions. J. Gambl. Stud..

[32] Lee S.J., Lee D.Y., Jeong E.J. (2016). The effects of gambling game play on adolescents’ morality—Focus on the personal trait. J. Korea Game Soc..

[33] Tremblay J., Stinchfield R., Wiebe J., Wynne H. (2010). Canadian Adolescent Gambling Inventory (CAGI) Phase III.

[34] Korea Center on Gambling Problems (2015). 2015 Survey on Youth Leisure and Culture.

[35] Kim E., Kwon S.J. (2022). The effect of speculative experience in internet games on adolescent gambling problems: Moderated mediating effect of gambling beliefs and attention control. Korean J. Youth Stud..

[36] Hayes A.F. (2018). Introduction to Mediation, Moderation, and Conditional Process Analysis: A Regression-Based Perspective.

[37] Kline T. (2005). Psychological Testing: A Practical Approach to Design and Evaluation.

[38] Kim H.Y. (2013). Statistical notes for clinical researchers: Assessing normal distribution (2) using skewness and kurtosis. Restor. Dent. Endod..

[39] Griffiths M.D. (2018). Is the buying of loot boxes in video games a form of gambling or gaming?. Gaming Law Rev..

[40] Park H.A., Lee J.J. (2017). Improving the regulation on the online stochastic game item. Korean J. Broadcast. Telecom Res..

[41] Lee J.K., Lee S.B. (2021). The influence of internet game speculative experience on gambling attitude among adolescents: Focusing on the mediation effects of sensitivity to reward level and irrational beliefs. Ment. Health Soc. Work.

[42] Jeon J.S., Kim K. The influence of contact about gambling on gambling addiction: The mediation effect of Irrational gambling belief. Proceedings of the Korean Psychol Assoc Annual Conference.

[43] Suhr J.A., Tsanadis J. (2007). Affect and personality correlates of the Iowa gambling task. Personal. Individ. Dif..

[44] Bandura A., Walters R.H. (1977). Social Learning Theory.

